# A common mechanism of clinical HIV-1 resistance to the CCR5 antagonist maraviroc despite divergent resistance levels and lack of common gp120 resistance mutations

**DOI:** 10.1186/1742-4690-10-43

**Published:** 2013-04-20

**Authors:** Michael Roche, Hamid Salimi, Renee Duncan, Brendan L Wilkinson, Kelechi Chikere, Miranda S Moore, Nicholas E Webb, Helena Zappi, Jasminka Sterjovski, Jacqueline K Flynn, Anne Ellett, Lachlan R Gray, Benhur Lee, Becky Jubb, Mike Westby, Paul A Ramsland, Sharon R Lewin, Richard J Payne, Melissa J Churchill, Paul R Gorry

**Affiliations:** 1 Center for Virology, Monash University, Melbourne, Victoria, Australia; 2 Center for Immunology, Monash University, Melbourne, Victoria, Australia; 3 Department of Microbiology, Monash University, Melbourne, Victoria, Australia; 4 Department of Infectious Diseases, Monash University, Melbourne, Victoria, Australia; 5 Department of Biochemistry and Molecular Biology, Monash University, Melbourne, Victoria, Australia; 6 Department of Medicine, Monash University, Melbourne, Victoria, Australia; 7 Department of Immunology, Monash University, Melbourne, Victoria, Australia; 8 Department of Microbiology and Immunology, University of Melbourne, Victoria, Australia; 9 Department of Surgery (Austin Health), University of Melbourne, Victoria, Australia; 10 School of Chemistry, The University of Sydney, New South Wales, Australia; 11 Department of Microbiology, Immunology, and Molecular Genetics, David Geffen School of Medicine, UCLA, Los Angeles, CA, USA; 12 Pfizer Global Research and Development, Sandwich, UK; 13 Infectious Diseases Unit, The Alfred Hospital, Melbourne, Victoria, Australia; 14 Present address: Fred Hutchinson Cancer Research Center, Seattle, WA, USA

**Keywords:** HIV-1, Maraviroc, Resistance, Env, gp120, V3 loop, CCR5 N-terminus, CCR5 ECLs

## Abstract

**Background:**

The CCR5 antagonist maraviroc (MVC) inhibits human immunodeficiency virus type 1 (HIV-1) entry by altering the CCR5 extracellular loops (ECL), such that the gp120 envelope glycoproteins (Env) no longer recognize CCR5. The mechanisms of HIV-1 resistance to MVC, the only CCR5 antagonist licensed for clinical use are poorly understood, with insights into MVC resistance almost exclusively limited to knowledge obtained from *in vitro* studies or from studies of resistance to other CCR5 antagonists. To more precisely understand mechanisms of resistance to MVC *in vivo*, we characterized Envs isolated from 2 subjects who experienced virologic failure on MVC.

**Results:**

Envs were cloned from subjects 17 and 24 before commencement of MVC (17-Sens and 24-Sens) and after virologic failure (17-Res and 24-Res). The Envs cloned during virologic failure showed broad divergence in resistance levels, with 17-Res Env exhibiting a relatively high maximal percent inhibition (MPI) of ~90% in NP2-CD4/CCR5 cells and peripheral blood mononuclear cells (PBMC), and 24-Res Env exhibiting a very low MPI of ~0 to 12% in both cell types, indicating relatively “weak” and “strong” resistance, respectively. Resistance mutations were strain-specific and mapped to the gp120 V3 loop. Affinity profiling by the 293-Affinofile assay and mathematical modeling using VERSA (Viral Entry Receptor Sensitivity Analysis) metrics revealed that 17-Res and 24-Res Envs engaged MVC-bound CCR5 inefficiently or very efficiently, respectively. Despite highly divergent phenotypes, and a lack of common gp120 resistance mutations, both resistant Envs exhibited an almost superimposable pattern of dramatically increased reliance on sulfated tyrosine residues in the CCR5 N-terminus, and on histidine residues in the CCR5 ECLs. This altered mechanism of CCR5 engagement rendered both the resistant Envs susceptible to neutralization by a sulfated peptide fragment of the CCR5 N-terminus.

**Conclusions:**

Clinical resistance to MVC may involve divergent Env phenotypes and different genetic alterations in gp120, but the molecular mechanism of resistance of the Envs studied here appears to be related. The increased reliance on sulfated CCR5 N-terminus residues suggests a new avenue to block HIV-1 entry by CCR5 N-terminus sulfopeptidomimetic drugs.

## Background

Human immunodeficiency virus type 1 (HIV-1) entry into cells is mediated by the viral envelope glycoproteins (Env) that decorate the surface of the virion (reviewed in [1,2]). The Env complex is organized into trimers, and consists of gp120, which is positioned on the surface of the viral membrane and presents the receptor binding surfaces of Env, and gp41, which is occluded by gp120 and anchors Env to the viral membrane. The primary point of contact between Env and the host cell is between gp120 and cellular CD4. This leads to structural rearrangements in gp120 that expose the binding site for a cellular coreceptor, which is either of the chemokine receptors CCR5 or CXCR4. Binding of gp120 to coreceptor leads to further structural changes in Env that expose the fusion peptide of gp41 that enables virus-cell fusion and entry of the virion core into the cell.

CCR5 comprises seven transmembrane helices that form a central hydrophobic pocket. Extending from the helices are the chemokine binding domains, comprising the N-terminus and three extracellular loops (ECL1, 2 and 3). Current models of gp120 binding to CCR5 suggest that the crown of the gp120 V3 loop interacts principally with the ECL2 region, while the gp120 bridging sheet that is formed between the C1, C2 and C4 domains of gp120 after CD4 binding, and the stem of the V3 loop interact with the CCR5 N-terminus [3-6]. Sulfation of tyrosine residues in the CCR5 N-terminus, particularly those at position 10 and 14, has been shown to be important for HIV-1 entry [7,8].

CCR5 antagonists belong to a relatively new class of HIV-1 antivirals known as entry inhibitors. They act by binding to the hydrophobic pocket in CCR5 formed by the transmembrane helices [9-14], which leads to structural alterations in the ECLs such that they are no longer recognized by gp120 [10,13-15]. The structure of the N-terminus does not appear to be affected by the binding of CCR5 antagonists to CCR5 [15]. Thus, CCR5 antagonists do not competitively block the binding of Env to CCR5, rather they are allosteric inhibitors of HIV-1 entry.

Maraviroc (MVC) [16,17] is the only CCR5 antagonist that has been licensed for clinical use as an HIV-1 antiretroviral therapy [18]. Other CCR5 antagonist HIV-1 inhibitors that are no longer being pursued for clinical development due to lack of clinical efficacy and/or adverse side effects include vicriviroc (VVC) [19], aplaviroc (APL) [20] and TAK-779 [21]. HIV-1 can develop clinical resistance to CCR5 antagonists by two routes. The first pathway is through emergence of minor CXCR4-using HIV-1 variants that were not detected in plasma prior to initiation of CCR5 antagonist therapy [22-29]. The second pathway to resistance is via continued use of CCR5, which is characterized not by shifts in IC_50_ (as would be expected for a competitive inhibitor), but rather by reductions in the maximal percent inhibition (MPI) [15,28,30-39]. Reductions in MPI are due to the resistant virus developing the ability to bind to the antagonist-modified form of CCR5 [34,35]. The plateau height of the MPI is also informative, with viruses that display a relatively high MPI (>80%) having relatively inefficient usage of the antagonist-modified form of CCR5 compared to unmodified CCR5, and viruses that display a relatively low MPI (<20%) having relatively efficient usage of the antagonist-modified form of CCR5 compared to unmodified CCR5.

Multiple studies have demonstrated that adaptive changes in the gp120 V3 loop are almost always responsible for the ability of resistant viruses to utilize the antagonist-modified form of CCR5 [15,32-34,37,40-42], although mutations in gp41 conferring this ability have also been observed [43-45]. No signature pattern of common amino acid mutations has been identified that would predict resistance to CCR5 antagonists, such as that which has been noted for HIV-1 resistance to the fusion inhibitor enfuviritide. Rather, the V3 loop alterations responsible for resistance to CCR5 antagonists appear to be strain specific [31,37,44,46,47].

The mechanisms underlying the ability of viruses with resistance to CCR5 antagonists to recognize the antagonist-modified form of CCR5 are incompletely understood. Most studies suggest that resistant viruses develop an increased reliance on the CCR5 N-terminus, most likely signaling a shift in gp120 binding to a region of CCR5 not modified by the binding of antagonist [15,32,42,48-50]. However, a recent study of VVC resistant strains showed heterogeneity in the ability of the resistant viruses to interact with VVC-modified CCR5, suggesting differing resistance mechanisms [38]. There is also evidence to suggest that resistant viruses must still interact with the drug modified ECLs, although the degree to which this interaction is required may be strain specific [15,51,52].

The vast majority of studies of HIV-1 resistance to CCR5 antagonists, however, have been conducted on resistant viruses that were generated *in vitro*[34,37,53,54], or on viruses isolated from subjects failing therapy by CCR5 antagonists that are no longer being pursued for clinical development, such as VVC, APL and TAK-779 [30,32,33,42]. In contrast, there is a paucity of data on MVC-resistant viruses arising *in vivo*[15,55]. Since different CCR5 antagonists cause subtly different structural alterations in CCR5 [14,15], it is unlikely that mechanisms of HIV-1 resistance to VVC, APL and TAK-779 can completely recapitulate those to MVC.

In this study we characterized the genetic, structural and functional properties of MVC-resistant Envs derived from two subjects who developed phenotypic resistance *in vivo*, with comparison to Envs derived from the same subjects prior to commencement of MVC. By measuring the MPIs in different cell types and by conducting quantitative Env-CCR5 affinity profiling studies, we show that the magnitude of MVC resistance was highly divergent in these subjects, and was characterized by either a highly efficient or a very inefficient ability of the resistant Envs to engage MVC-bound CCR5. Despite highly divergent resistance phenotypes and a lack of common gp120 resistance mutations, both resistant Envs exhibited an almost superimposable pattern of dramatically increased reliance on sulfated tyrosine residues in the CCR5 N-terminus, and on histidine residues in the CCR5 ECLs. Furthermore, neither of the MVC-resistant Envs displayed broad cross resistance to other CCR5 antagonists. Together, our results suggest that clinical resistance to MVC may involve divergent Env phenotypes and different genetic alterations in gp120, but the molecular pathway to resistance for the Envs studied here appears to be related.

## Results and discussion

### Characteristics of the MVC sensitive and MVC resistant Env clones

**Figure 1:**
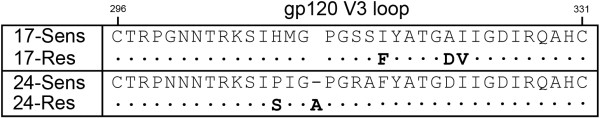
**V3 loop amino acid alterations responsible for resistance to MVC by viruses harbored by subjects 17 and 24.** The V3 loop alterations shown in bold face were demonstrated to be associated with resistance to MVC by 17-Res and 24-Res Envs [47,56]. Dots indicate residues identical to the parental sensitive Envs, and dashes indicate gaps. The numbering is based of the HXB2 Env amino acid sequence.

Our previous studies revealed the molecular mechanisms responsible for the recognition of the MVC-modified form of CCR5 by a MVC-resistant Env that was derived *in vitro*[51]. To determine how these mechanisms relate to those occurring *in vivo*, we obtained HIV-1 Envs derived from two subjects (referred to as subjects 17 and 24) who commenced MVC treatment along with optimized background therapy as part of the MOTIVATE phase III clinical trial, and who experienced virologic failure associated with phenotypically-verified MVC resistance [47,52,56,57]. Envs were isolated from plasma before commencement of MVC treatment (referred to as 17-Sens and 24-Sens Envs) and after virologic failure (referred to as 17-Res and 24-Res Envs) [57]. These Envs are representative of the dominant circulating HIV-1 variants [52,56]. When subcloned into the pSVIII-Env expression vector and used to produce Env-pseudotyped luciferase reporter viruses, all 4 Envs were shown to be specific for CCR5 in single round entry assays with NP2-CD4/CCR5 and NP2-CD4/CXCR4 cells, with no evidence of specificity for CXCR4 detected (data not shown). The Envs are also predicted to be of R5 phenotype by the Geno-2-Pheno coreceptor usage prediction algorithm. Env sequence analysis showed amino acid changes in the gp120 V3 loop that distinguished the resistant Envs from the parental sensitive Envs (Figure 1). Of note, 24-Res Env contains an insertion within the V3 loop GPG crown, which is unusual for R5 Envs.

### The MVC resistant Envs are highly divergent in their level of resistance

**Figure 2:**
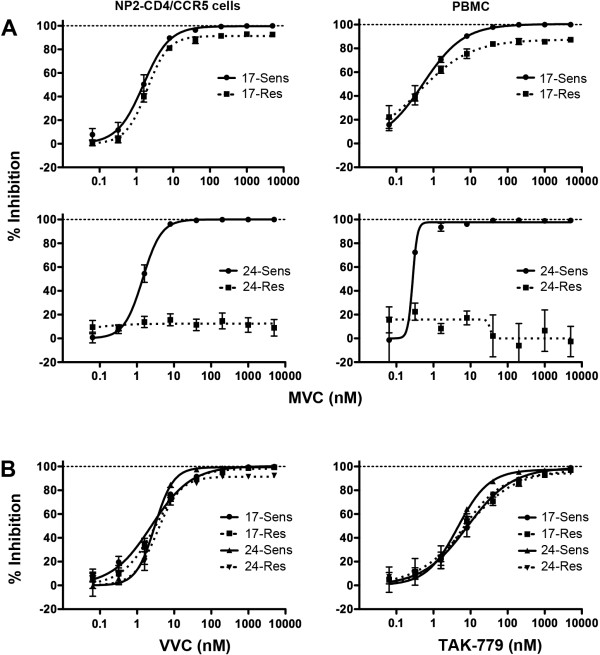
**Profiles of resistance to MVC and cross-resistance to other CCR5 antagonists.** Luciferase reporter viruses pseudotyped with Envs from subject 17 or subject 24 were used to infect NP2-CD4/CCR5 (**A**, left panels) or PBMC (**A**, right panels) in the presence of increasing concentrations of MVC. The same virus preparations were used to infect NP2-CD4/CCR5 cells in the presence of increasing concentrations of VVC or TAK-779 (**B**). Virus inhibition curves were constructed as described in the Methods. The data points represent the mean and standard error of the mean from quadruplicate wells, and are the results from 5 (MVC) or 3 (VVC and TAK-779) independent experiments. The independent PBMC experiments were performed in cells obtained from different donors. Inhibition curves were constructed using Prism, version 4.0c (GraphPad).

To compare the magnitude of resistance between 17-Res and 24-Res Envs, we first assessed their resistance profiles and measured their MPIs in NP2-CD4/CCR5 cells and PBMC when pseudotyped onto luciferase reporter viruses (Figure 2A). In both cell types 17-Sens and 24-Sens Envs were completely inhibited by MVC, as demonstrated by MPIs of ~100%, whilst 17-Res and 24-Res Envs were incompletely inhibited by MVC. The extent of incomplete inhibition of 17-Res and 24-Res Envs by MVC was variable, with 17-Res Env displaying a relatively high MPI in NP2-CD4/CCR5 cells (91.4 ± 1.3%) and PBMC (87.4±2.4%), and 24-Res displaying a very low MPI in NP2-CD4/CCR5 cells (12.5 ± 2.5%) (Table 1). 24-Res Env was completely insensitive to MVC inhibition in PBMC, as shown by the lack of an obtainable dose response curve (Figure 2A). These results suggest that 17-Res Env has a relatively “weak” MVC resistance phenotype, whereas 24-Res Env has a relatively “strong” MVC resistance phenotype. The results further suggest that resistance to MVC in HIV-1 infected subjects may present in different ways and may potentially have different clinical consequences. These MVC resistant Envs, which display a wide divergence in resistance levels, therefore offer a unique opportunity to better understand alternative mechanisms of MVC resistance that may arise *in vivo*.

**Table 1:** MPIs of the HIV-1 Envs to MVC, VVC and TAK-779

**Env**	**MVC**				**VVC**				**TAK-779**			
	**MPI**	**SEM**	**95% CI**	**Resistance phenotype**	**MPI**	**SEM**	**95% CI**	**Resistance phenotype**	**MPI**	**SEM**	**95% CI**	**Resistance phenotype**
**17-Sens**	99.7	2.1	99.5-103.9	Sensitive	100.5	2.0	96.2-104.7	Sensitive	99.2	2.6	93.7-104.7	Sensitive
**17-Res**	91.4	1.3	88.7-94.1	Weakly Resistant	98.3	1.9	94.4-102.2	Sensitive	97.2	5.4	85.9-108.5	Sensitive
**24-Sens**	100.1	1.5	97.1-103.1	Sensitive	99.4	2.0	95.2-103.6	Sensitive	97.5	1.6	94.2-100.7	Sensitive
**24-Res**	12.5	2.5	7.3-17.6	Strongly Resistant	91.4	2.2	86.9-96.0	Weakly Cross-resistant	94.7	2.3	89.9-99.5	Weakly Cross-resistant

MPIs were determined in NP2-CD4/CCR5 cells, and are calculated from 3 (VVC, TAK-779) or 5 (MVC) independent experiments.

MPI, maximal percent inhibition; SEM, standard error of the mean; CI, confidence interval; MVC, maraviroc; VVC, vicriviroc.

### MVC resistance by 17-Res and 24-Res Envs is not associated with broad cross resistance to other CCR5 antagonists

Although MVC is the only CCR5 antagonist approved for clinical use, other CCR5 antagonists are presently in the developmental pipeline, so whether viruses that are resistant to MVC are cross resistant to other CCR5 antagonists remains an important question. We therefore next determined the sensitivity of the panel of Envs to inhibition by VVC and TAK-779 in NP2-CD4/CCR5 cells (Figure 2B) (Table 1). As expected, both 17-Sens and 24-Sens Envs were completely inhibited by both VVC and TAK-779, as demonstrated by MPIs of ~100% by both CCR5 antagonists. 17-Res Env was also completely inhibited by VVC (MPI 98.3 ± 1.9%) and TAK-779 (MPI 97.2 ± 5.4%), indicating that this “weakly” MVC resistant 17-Res Env is not cross resistant to these other CCR5 antagonists. Although 24-Res Env was strongly resistant to MVC with MPIs ~12% in NP2-CD4/CCR5 cells (Figure 2A), it showed comparatively very modest levels of cross resistance to VVC (91.4 ± 2.2%) and TAK-779 (94.7 ± 2.3%). These results show that despite divergent levels of MVC resistance by 17-Res and 24-Res Envs, cross-resistance to other CCR5 antagonists was either absent or only weakly evident.

Broad cross resistance to multiple CCR5 antagonists as a consequence of HIV-1 evolving resistance to one particular CCR5 antagonist is relatively common for viral strains that have developed resistance to VVC [30,33,41,53], APL [39] and AD101 [40] which is a preclinical precursor to VVC [13]. In contrast, strains that have evolved resistance to MVC have shown either no cross resistance to other CCR5 antagonists [34,51] or narrow cross resistance [15], regardless of whether resistance was generated *in vitro* or *in vivo*. These results, in conjunction with our new data (Figure 2B), suggest that a lack of appreciable cross resistance or a narrow cross resistance profile may be unique to HIV-1 strains that have evolved resistance to MVC. It is known that different CCR5 antagonists modify the structure of CCR5 in subtly different ways [15]. Therefore, whilst acquisition of the ability of HIV-1 to recognize the VVC-, AD101-, or APL-modified forms of CCR5 can allow recognition of other antagonist-modified forms of CCR5, acquisition of the ability of HIV-1 to recognize the MVC-modified form of CCR5 does not appear to lend itself to cross resistance. Whilst these observations suggest that HIV-1 that has developed resistance to MVC may remain susceptible to new CCR5 antagonists should they eventually reach the clinic, they underscore the argument that the study of HIV-1 resistance to other CCR5 antagonists most likely does not precisely predict mechanisms of resistance to MVC.

### Strain-specific V3 loop alterations contribute to MVC resistance

**Figure 3:**
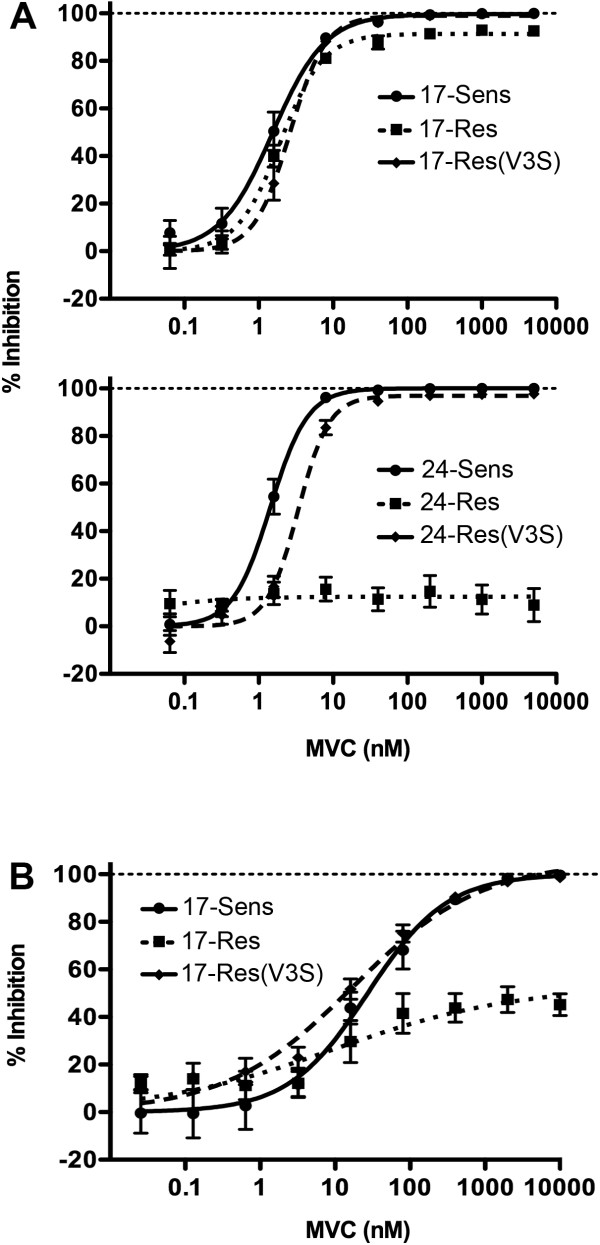
**V3 loop alterations contribute to MVC resistance in subjects 17 and 24.** Luciferase reporter viruses pseudotyped with unmodified Envs from subject 17 or subject 24, or resistant Envs carrying the V3 loop region of the respective sensitive Envs, were used to infect NP2-CD4/CCR5 cells in the presence of increasing concentrations of MVC (**A**). A similar experiment with unmodified and mutant Envs from subject 17 was conducted in 293-Affinofile cells that were induced to express maximal levels of CD4 and CCR5 as described previously [51,58] (**B**). Virus inhibition curves were constructed as described in the Methods. The data points represent the mean and standard error of the mean from quadruplicate wells, and are the results from 4 independent experiments. Inhibition curves were constructed using Prism, version 4.0c (GraphPad).

To better understand whether the V3 loop alterations associated with MVC resistance in subjects 17 and 24 contribute to the resistance phenotypes (Figure 1), we next produced mutants of 17-Res and 24-Res that contained the V3 loop alterations of 17-Sens and 24-Sens Envs, respectively. We refer to these mutants as 17-Res(V3S) and 24-Res(V3S), respectively. The sensitivity of these Env mutants to MVC was first tested in NP2-CD4/CCR5 cells, with comparison to the unmodified Envs (Figure 3A). 17-Res(V3S) was completely inhibited by MVC in NP2-CD4/CCR5 cells, as demonstrated by an MPI of 99.03 ± 1.85% (95% CI 95.25 – 102.8), thus indistinguishable from the phenotype of 17-Sens Env and significantly different to that of the parental 17-Res Env (Table 1). 24-Res(V3S) was also completely inhibited by MVC, as demonstrated by an MPI of 96.94 ± 1.51% (95% CI 93.85 – 100.0). These results suggest that the V3 loop alterations shown in Figure 1 are necessary for MVC resistance in subjects 17 and 24.

Because the MVC resistance phenotype of 17-Res Env was only subtly demonstrated in NP2-CD4/CCR5 cells, we next repeated the MVC inhibition studies of 17-Sens, 17-Res and 17-Res(V3S) Envs in 293-Affinofile cells induced to maximally express CD4 and CCR5, with the rationale that MVC resistance phenotypes are usually more pronounced in cell lines that express higher levels of CCR5 [36,58] (Figure 3B). 17-Sens Env was completely inhibited by MVC in the Affinofile cells, as demonstrated by an MPI of 100.1 ± 5.10% (95% CI 89.70 – 110.5). In contrast, 17-Res Env was incompletely inhibited, with an MPI of 53.0 ± 9.56% (95% CI 33.53 – 72.47). The substantially lower MPI to MVC by 17-Res Env in Affinofile cells compared to NP2-CD4/CCR5 cells confirms that 17-Res Env is genuinely MVC resistant. 17-Res(V3S) was completely inhibited by MVC in Affinofile cells as demonstrated by an MPI of 105.5 ± 4.96 (95% CI 95.43 – 115.6), providing further evidence that the V3 loop alterations in 17-Res Env are important for MVC resistance in this subject.

### The magnitude of MVC resistance is determined by the efficiency of the interaction with MVC-modified CCR5

The preceding studies suggest that 17-Res Env, which has a comparatively high MPI to MVC, has a relatively inefficient ability to recognize MVC-modified CCR5, but that 24-Res Env, which has a comparatively low MPI to MVC, has a very efficient ability to do so. We therefore next used the 293-Affinofile affinity profiling system [59,60] to quantify the ability of 17-Res and 24-Res Envs to engage MVC-bound CCR5. In this system, CD4 and CCR5 expression is controlled by separate inducible promoters, permitting independent variation of CD4 and CCR5 expression over a physiological concentration range [59]. When 48 differentially-induced cell populations are subjected to single-round entry assays with Env-pseudotyped luciferase reporter viruses and data sets quantitatively analyzed by mathematical modeling using the VERSA (Viral Entry Receptor Sensitivity Analysis) computational platform [59,60], vector metrics are generated; vector angles measure the degree of CD4- and CCR5-dependence, and vector magnitude measures the efficiency of virus entry. These quantitative data can be used to dissect gp120-CD4/CCR5 interactions. For example, viruses displaying relatively low vector angles and relatively high vector magnitudes are typical of those that have a more efficient interaction with CCR5 [39,42,51].

**Figure 4:**
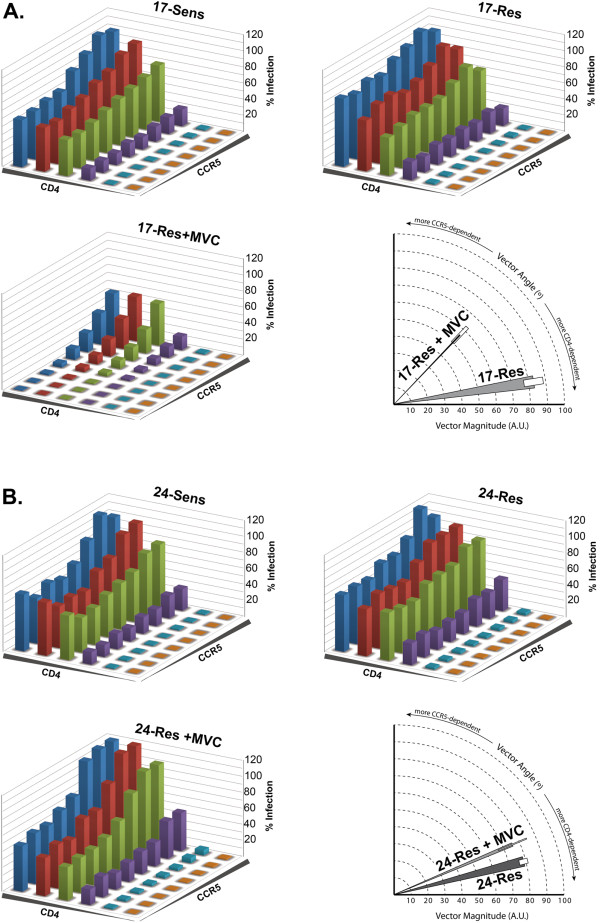
**The level of resistance to MVC is dependent on the efficiency of the interaction with MVC-occupied CCR5.** 48 differentially induced 293-Affinofile cell populations were produced as described in Methods, and were infected with luciferase reporter viruses pseudotyped with Envs from subject 17 (**A**) or subject 24 (**B**). Infections of viruses pseudotyped with the MVC-resistant 17-Res and 24-Res Envs were also done in the presence of 10 µM MVC (lower left panels). For infections in the absence of drug, virus entry levels were normalized to entry achieved in cells expressing the highest levels of CD4 and CCR5. For infections in the presence of drug, virus entry levels were normalized to entry achieved in cells expressing the highest levels of CD4 and CCR5 in the absence of drug, as described previously [51]. 17-Sens and 24-Sens Envs were completely inhibited by MVC in the 293-Affinofile populations (data not shown). The data shown are means of duplicate infections and are representative of three independent experiments. Shaded wedges along each axis represent increasing concentrations of CD4 and CCR5, as indicated in the Methods. VERSA metrics were calculated for 17-Res (**A**, lower right panel) and 24-Res Env (**B**, lower right panel) in the absence and presence of drug, as described in Methods. The shaded wedges represent the SEM of the vector angles, and the boxes represent the SEM of the vector magnitudes.

The results of single round entry assays in 293-Affinofile cells showed that in the absence of MVC, the sensitive and resistant Envs from both subjects had remarkably similar infectivity profiles; they were highly sensitive to alterations in CD4 levels but much less sensitive to alterations in CCR5 levels (Figure 4A,B). In fact, at the lowest CCR5 expression level, these Envs achieved ~50 to ~80% of maximal entry when moderate to high levels of CD4 were present, suggesting that they all have a relatively efficient and equivalent ability to interact with unmodified CCR5. However, in the presence of MVC, the “weakly” resistant 17-Res Env was highly sensitive to alterations in both CD4 and CCR5 levels, and even at high levels of both receptors 17-Res could only achieve ~50% of the maximal entry achieved when MVC was absent (Figure 4A). In contrast, in the presence of MVC, the “strongly” resistant 24-Res Env showed an infectivity profile similar to that which was achieved in the absence of MVC (Figure 4B). In these cell populations, MVC was able to inhibit the entry of 17-Sens and 24-Sens Envs, and inhibited the entry of other MVC-sensitive R5 Envs that have highly efficient CCR5 usage such as ADA, YU2, NB6-C3 and NB8-C2 Envs [61,62] (data not shown). Together, these results show that 17-Res Env interacts with MVC-modified CCR5 inefficiently, whereas 24-Res Env interacts with MVC-modified CCR5 relatively efficiently.

These results are reflected quantitatively by the VERSA vector metrics. In the absence of MVC, 17-Sens and 17-Res Envs had similar vector angles of 11.2 ± 1.1° and 9.0 ± 1.5°, respectively. 24-Sens and 24-Res Envs also had similar vector angles of 12.9 ± 0.8° and 14.4 ± 1.2°, respectively, in the absence of MVC. These vector angles are well below the range of what is normally observed for subtype B HIV-1 Envs using this system (30 to 60°) [59,60], indicating highly efficient interactions with unmodified CCR5. In contrast, the “weakly” resistant 17-Res Env had significantly higher vector angles of 46.4 ± 0.2° for recognition of MVC-bound CCR5 (p < 0.0001 by an unpaired *t* test), and significantly lower vector magnitudes which are a measure of overall viral infectivity (p = 0.048 by an unpaired *t* test) (Figure 4A, bottom right panel). The “strongly” resistant 24-Res Env had much less pronounced increases in vector angles of 22.9 ± 0.5° for recognition of MVC-bound CCR5, yet these increases were statistically significant (p = 0.0027 by an unpaired *t* test) (Figure 4B, bottom right panel). However, 24-Res Env showed no change in vector magnitudes for recognition of MVC-bound CCR5, indicating no overall reduction in viral infectivity.

Together, the 293-Affinofile infectivity profiles and VERSA metrics suggest that the development of HIV-1 resistance to MVC in subject 17 compromised the efficiency of interaction between gp120 and CCR5, whereas the development of resistance in subject 24 did not. When viewed in the context of the different MPIs to MVC by these resistant strains, our data illustrate highly divergent MVC resistance phenotypes by these two HIV-1 variants that developed *in vivo*. Our results are in agreement with those of other studies which suggested that the magnitude of HIV-1 resistance to CCR5 antagonists is dictated by the ability of resistant viruses to recognize antagonist bound CCR5 [15,36,42,51]. In other words, the more efficiently the resistant Env can recognize the drug-receptor complex compared to free receptor, the greater the level of resistance. Our results further show, in two examples of highly divergent resistance levels that developed *in vivo*, that there was no change in CD4 dependence between the MVC-sensitive and MVC-resistant Envs, which has been previously reported for a MVC-resistant Env derived *in vitro*[63]. Changes in gp120 binding to CD4 are therefore not likely to be major pathway to MVC-resistance in subjects failing therapy.

### Both 17-Res and 24-Res Envs adopt an increased dependence on the CCR5 N-terminus to escape MVC inhibition

Previous studies have shown that escape from CCR5 antagonists by resistant viruses that were derived either *in vitro*[48,50,51] or *in vivo*[15,32,38,42] involves HIV-1 adopting an increased reliance on the CCR5 N-terminus, presumably because this region of CCR5 is not structurally modified by CCR5 antagonists [15]. The extent to which this occurs in HIV-1 strains that have divergent MVC resistance levels, however, is unknown.

**Figure 5:**
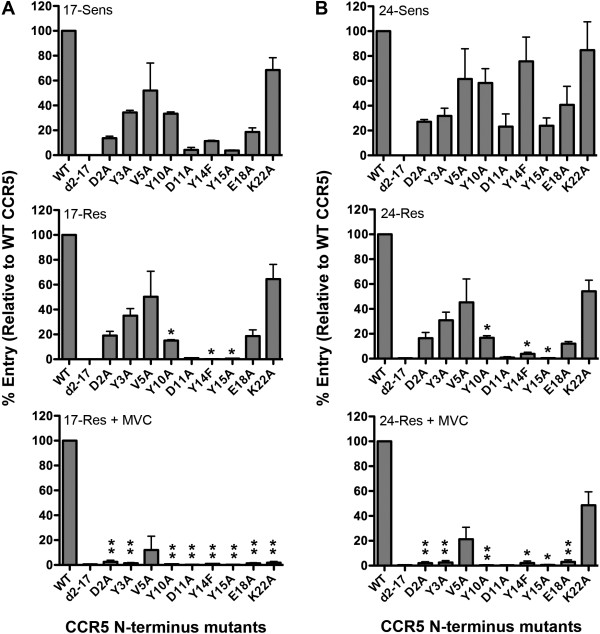
**17-Res and 24-Res Envs exhibit a similar increased dependence on the CCR5 N-terminus.** Luciferase reporter viruses pseudotyped with Envs from subject 17 (**A**) or 24 (**B**) were used to infect NP2-CD4 cells expressing equivalent levels of WT CCR5 or CCR5 with mutations in the N-terminus region. Infections were performed in the absence or presence of 10 µM MVC. Viral entry in cells expressing CCR5 mutants was normalized to entry obtained in cells expressing an equivalent amount of WT CCR5. Compared to background levels of entry by virus pseudotyped with a non-functional Env [66] (~1,300 RLU), the entry levels of 17-Res and 24-Res in cells expressing WT CCR5 the presence of MVC were approximately 60- and 380-fold higher, respectively (~80,000 and 500,000 RLU, respectively). The data shown are the means and SEM from a compilation of 3 independent experiments. * p<0.05 for increases in the dependence on a particular residue for the resistant Envs compared to the respective parental sensitive Env. **p<0.05 for increases in the dependence on a particular residue compared to respective resistant Envs in the absence of drug (unpaired t-test).

We therefore next determined the mechanism of engagement between 17-Res and 24-Res Envs and MVC-modified CCR5. Single round entry assays were conducted in NP2-CD4 cells expressing either wild type (WT) CCR5 or CCR5 containing various mutations in the N-terminal region using Env-pseudotyped luciferase reporter viruses, in the presence or absence of MVC (Figure 5). The levels of virus entry in cells expressing CCR5 mutants were expressed as percentages of that attained in cells expressing equivalent levels of WT CCR5, which was verified by flow cytometry as described previously [62,64,65] (data not shown). In the absence of MVC, both 17-Res and 24-Res Envs displayed significantly increased dependence on Tyr10, Tyr14 and Tyr15 residues in the CCR5 N-terminus, which are known to be sulfated *in vivo*, compared to their MVC-sensitive parental strains. Furthermore, in the presence of MVC when the resistant Envs are “forced” to interact with the drug-modified CCR5 to enter cells, both 17-Res and 24-Res Envs showed a critical reliance not only on Tyr10, Tyr14 and Tyr15, but also on Asp2, Tyr3, Asp11 and Glu18 residues in the CCR5 N-terminus. 17-Res Env adopted an increased reliance on Lys22 for recognition of MVC-bound CCR5, whereas 24-Res Env did not. Neither Env adopted an increased reliance on Val5.

These results demonstrate a shift to an increased reliance on the CCR5 N-terminus by both MVC-resistant Envs for interaction with MVC-modified CCR5. Strikingly, the pattern of increased reliance on individual CCR5 N-terminus residues by 17-Res and 24-Res Envs, in the absence and presence of MVC, was almost superimposable despite widely divergent resistance levels and widely divergent efficiencies for interaction with MVC-modified CCR5. This common pattern of increased reliance on CCR5 N-terminus residues is also remarkably similar to that which we have previously reported for a MVC-resistant Env that was generated *in vitro*[51], and very similar to that which has been previously reported for a MVC-resistant virus that developed *in vivo*[15]. Our results illustrate a common molecular pathway to altered usage of the CCR5 N-terminus as a means for the two resistant viruses studied to escape MVC, despite divergent resistance phenotypes and a lack of common gp120 resistance mutations.

### Increased reliance on the CCR5 N-terminus renders MVC-resistant Envs sensitive to neutralization by synthetic, sulfated peptide fragment of the CCR5 N-terminus

Increased reliance on the CCR5 N-terminus for interaction with MVC-bound CCR5 by the resistant Envs, particularly the increased reliance on the potentially sulfated Tyr10, Tyr14 and Tyr15 residues, suggests that this altered mode of HIV-1 entry into cells may be more susceptible to being neutralized by sulfated peptide fragments of the CCR5 N-terminus. We therefore produced a sulfated peptide corresponding to amino acids 1 to 22 of the human CCR5 N-terminus (sCCR5_1-22_). Although previous studies have employed mammalian expression systems to incorporate sulfate groups to peptides via the activity of cellular tyrosylprotein sulfotransferases [67-69], with this methodology sulfation cannot be regulated nor can it be incorporated in a homogenous manner, as demonstrated by the failure to achieve sulfation at Tyr15 [69]. To alleviate these limitations, we utilized a site-selective solid-phase synthetic approach that permits the high yielding production of sulfated peptides with high purity (>98%), and which are homogenously sulfated at defined tyrosine residues [70-72]. This relatively new methodology was used to produce a synthetic peptide fragment of the CCR5 N-terminus, sCCR5_1-22_, that was sulfated at Tyr10, Tyr14 and Tyr15.

**Figure 6:**
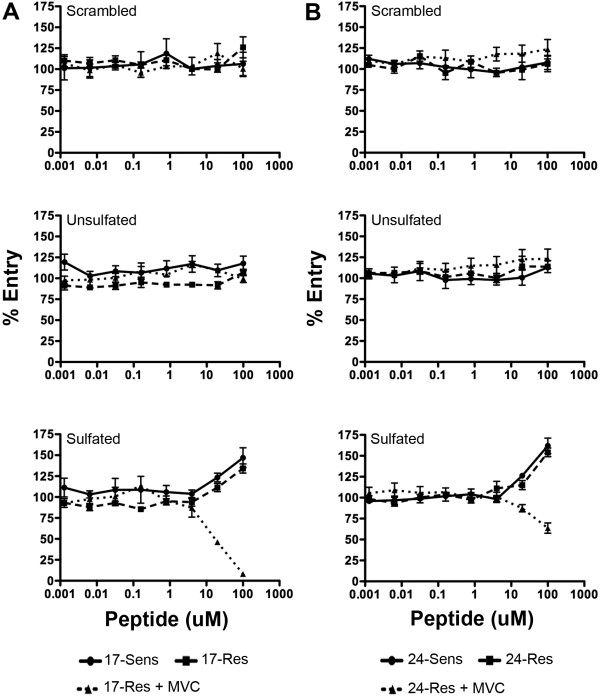
**A sulfated mimic of the CCR5 N-terminus can inhibit entry of MVC-resistant Envs.** NP2-CD4/CCR5 cells were preincubated with increasing concentrations of scrambled, unsulfated or sulfated sCCR5_1-22_ peptide prior to infection with luciferase reporter viruses pseudotyped with Envs from subject 17 (**A**) or 24 (**B**). Infections of viruses pseudotyped with 17-Res and 24-Res Envs were also performed in the presence of 10 µM MVC. The data are expressed as the percentage entry of that obtained in cells incubated with no inhibitor. Data points shown are the mean and SEM of triplicates, and are representative of three independent experiments.

We first confirmed that the synthetic sCCR5_1-22_ peptide could completely displace the binding of CD4-bound gp120 to the 17b gp120 mAb in a dose dependent manner using an ELISA (data not shown), demonstrating that sCCR5_1-22_ can bind to CD4-induced gp120 epitopes. We next tested the ability of sCCR5_1-22_ to inhibit the entry of the MVC-sensitive and MVC-resistant Envs in the presence or absence of MVC (Figure 6). As controls we used an unsulfated version of sCCR5_1-22_, and an unsulfated peptide that was composed of the same amino acids as sCCR5_1-22_ but in a scrambled order. The unsulfated and scrambled peptides had no affect on the infectivity of any of the Envs tested, regardless of whether MVC was present or absent. The sCCR5_1-22_ peptide was not capable of inhibiting the infectivity of any of the Envs in the absence of MVC, and indeed, they showed some enhancement of infectivity when the MVC-resistant and MVC-sensitive Envs were permitted to interact with unmodified CCR5. This enhancement of infectivity may represent some “pre-triggering” of gp120 to facilitate entry into cells via a favored interaction with the CCR5 ECLs. However, sCCR5_1-22_ could inhibit the entry of 17-Res (Figure 6A) and 24-Res Envs (Figure 6B) in the presence of MVC, when these Envs were forced to interact with MVC-modified CCR5 via an increased reliance on the CCR5 N-terminus.

**Figure 7:**
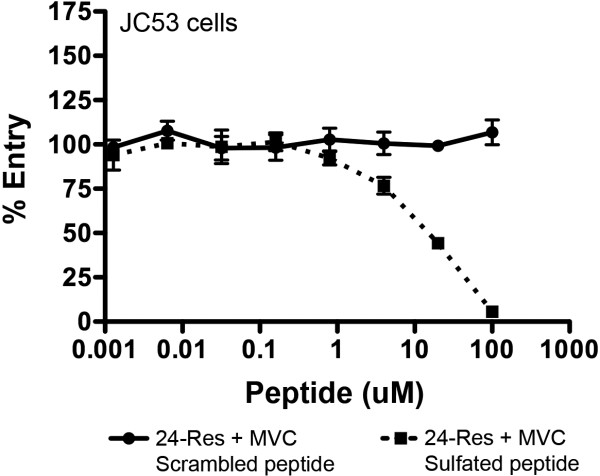
**Inhibition of 24-Res Env by sCCR5**_**1-22 **_**is more pronounced in cells expressing lower levels of CCR5.** JC53 cells, which express substantially lower levels of CCR5 than NP2-CD4/CCR5 cells [58], were infected with luciferase reporter viruses pseudotyped with 24-Res Env in the presence of 10 µM MVC and increasing concentrations of either scrambled peptide or sulfated sCCR5_1-22_ peptide. The data are expressed as the percentage entry of that obtained in cells incubated with no inhibitor. The data points shown are the mean and SEM of triplicates, and are representative of three independent experiments.

Interestingly, the “weakly” resistant 17-Res Env could be completely inhibited by 100 μM sCCR5_1-22_ in NP2-CD4/CCR5 cells, whereas the “strongly” resistant 24-Res Env was only partially inhibited. Because 24-Res Env has a more efficient interaction with MVC-bound CCR5 than 17-Res Env (see Figure 4), we hypothesized that 24-Res Env has increased affinity for the CCR5 N-terminus in the presence of MVC than 17-Res Env, which was outcompeting sCCR5_1-22_. To test this hypothesis we repeated the experiment in JC53 cells, which express substantially lower levels of CCR5 than NP2-CD4/CCR5 cells [58]. We reasoned that reducing the amount of available native CCR5 N-terminus would lead to greater inhibition of 24-Res Env in the presence of MVC by sCCR5_1-22_, and in this cell line we showed that 24-Res could be completely inhibited by 100 µM sCCR5_1-22_ (Figure 7). These results suggest that the inhibitory mechanism of sCCR5_1-22_ is competitive, and that 17-Res and 24-Res Envs have different affinities for the CCR5 N-terminus when interacting with MVC-bound CCR5. However, further inhibition studies in JC53 cells with 24-Sens Env in the absence of MVC are required to confirm this assertion. Our results also confirm that sulfation of the CCR5 N-terminus is critical for interaction with gp120 [5].

These results are consistent with the those of previous studies which showed that an antibody directed to the CCR5 N-terminus (CTC5) was able to neutralize the infectivity of viruses resistant to CCR5 antagonists, but not sensitive viruses [32,49,50], confirming the importance of interactions with the CCR5 N-terminus as a route for HIV-1 escape from CCR5 antagonists. Moreover, these results suggest a new avenue for development of HIV-1 therapeutics that could be useful for treating patients with resistance to CCR5 antagonists, or which could prevent resistance from occurring if co-administered with CCR5 antagonists. Whilst 100 µM concentrations are certainly not favorable for any peptide inhibitor, proof of principal is demonstrated here, and potency may potentially be increased by further development of sulfopeptidomimetic compounds based on the CCR5 N-terminus.

### Both 17-Res and 24-Res Envs adopt an altered mechanism of interaction with the CCR5 ECLs to escape MVC inhibition

**Figure 8:**
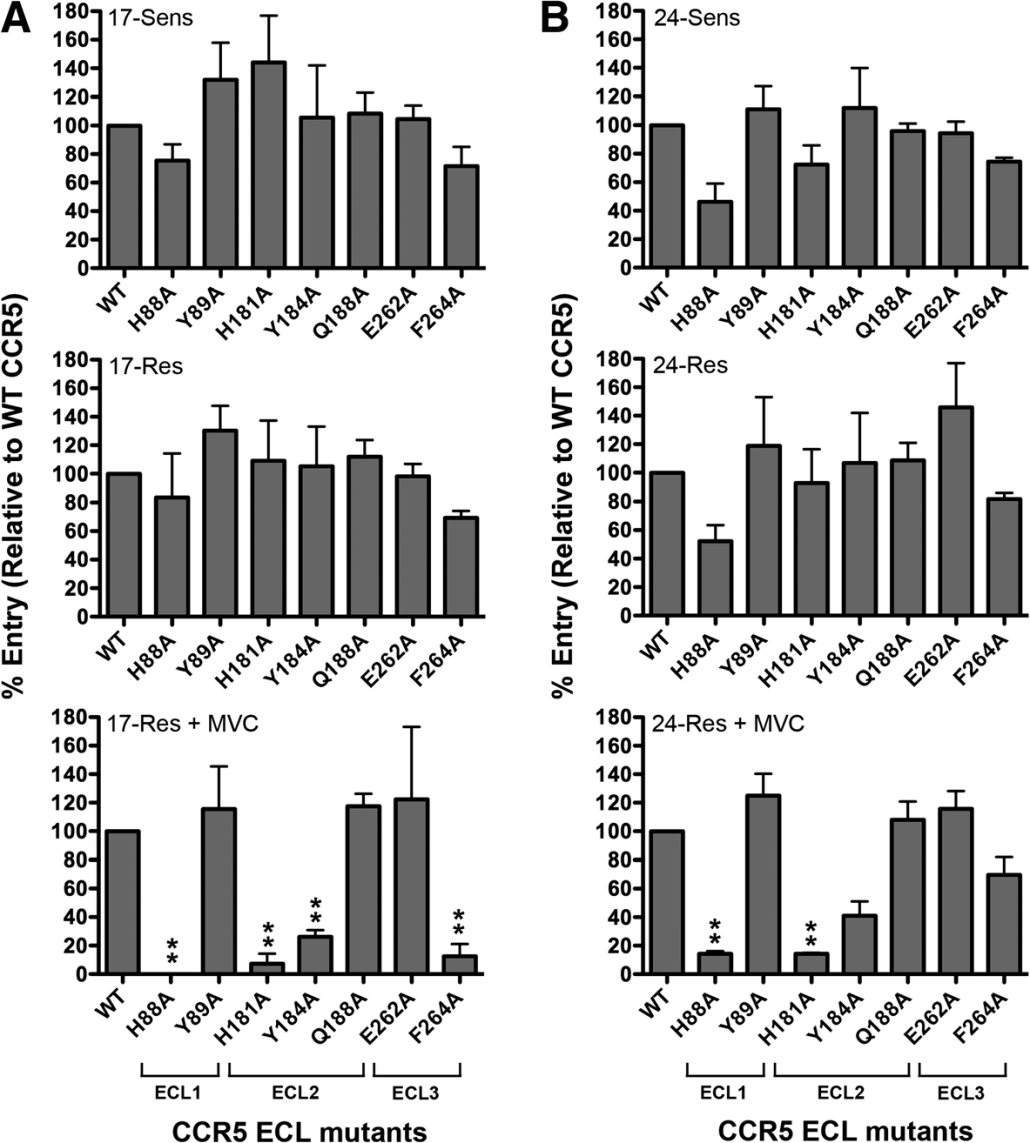
**17-Res and 24-Res Envs exhibit a similar altered recognition of the CCR5 ECLs.** Luciferase reporter viruses pseudotyped with Envs from subject 17 (**A**) or 24 (**B**) were used to infect NP2-CD4 cells expressing equivalent amounts of WT CCR5 or CCR5 with mutations in the ECL1, ECL2 and ECL3 regions. Infections were performed in the absence or presence of 10 µM MVC. Viral entry in cells expressing CCR5 mutants was normalized to entry obtained in cells expressing an equivalent amount of WT CCR5. In these experiments, MVC completely inhibited the entry of 17-Sens and 24-Sens Envs, regardless of the Env mutant tested (data not shown). The data shown are the means and SEM from a compilation of 3 independent experiments. **p<0.05 for increases in the dependence on a particular residue compared to respective resistant Envs in the absence of drug (unpaired t-test).

HIV-1 strains that evolved resistance to MVC *in vitro* or *in vivo* and display a narrow cross resistance profile have been shown to remain dependent on the MVC-modified CCR5 ECLs for entry [15,51]. To determine whether this is the case for HIV-1 strains that have divergent MVC resistance levels, we next conducted single round entry assays in NP2-CD4/CCR5 cells expressing equivalent levels of either WT CCR5 or CCR5 containing various mutations in the ECL1, ECL2 or ECL3 region using Env-pseudotyped luciferase reporter viruses, in the presence or absence of MVC. HIV-1 entry levels were measured and expressed as described above. In the absence of MVC, both 17-Res and 24-Res displayed similar profiles of dependence on these regions as compared to their parental sensitive Envs, at least with the CCR5 mutants tested (Figure 8). However, in the presence of MVC, 17-Res and 24-Res Envs both became critically reliant on His88 and His181 in the CCR5 ECL1 and ECL2 regions, respectively, which is similar to the pattern of increased reliance on these residues that we have reported for a MVC-resistant strain that was generated *in vitro*[51]. 17-Res Env also adopted an increased reliance on Tyr184 and Phe264 in the CCR5 ECL2 and ECL3 regions, respectively, whereas 24-Res Env did not. Thus, in addition to adopting a common mechanism of increased reliance on the CCR5 N-terminus (see Figure 5), MVC-resistant Envs also appear to adopt a common pattern of increased reliance on histidine residues in the CCR5 ECL1 and ECL2 regions, despite divergence in resistance levels and differing efficiencies for recognition of the drug-modified ECLs (see Figure 4).

**Figure 9:**
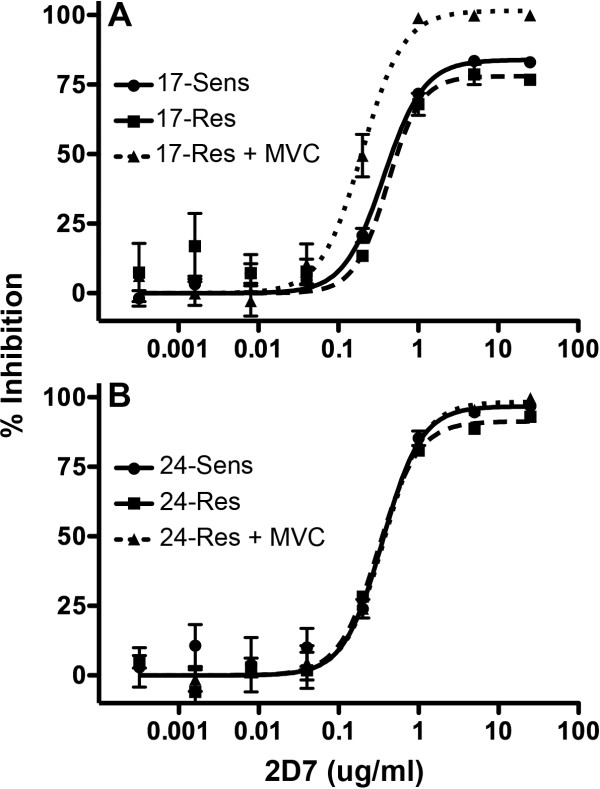
**MVC-resistant Envs remain sensitive to inhibition by the CCR5 ECL2-directed antibody 2D7.** NP2-CD4/CCR5 cells were preincubated with increasing concentrations of 2D7 prior to infection with luciferase reporter viruses pseudotyped with Envs from subject 17 (**A**) or 24 (**B**). Infections with 17-Res and 24-Res Envs were also performed in the presence of 10 µM MVC. The data are expressed as percentage inhibition, where inhibition in untreated cells was set to zero. The data shown are the mean and SEM of three independent experiments. Inhibition curves were constructed using Prism, version 4.0c (GraphPad).

To confirm that 17-Res and 24-Res Envs maintained a critical interaction with the MVC-modified CCR5 ECLs for HIV-1 entry into cells, we next performed virus inhibition assays in NP2-CD4/CCR5 cells using the 2D7 mAb, which is directed against the ECL2 region of CCR5 (Figure 9). All the Envs remained sensitive to inhibition by 2D7 when HIV-1 entry proceeded either via unmodified CCR5 or MVC-bound CCR5, confirming that both 17-Res and 24-Res still require contact with the MVC-modified CCR5 ECLs to escape MVC. These results are consistent with those of another study of a MVC-resistant strain that was generated *in vivo*, which also showed a requirement of MVC-resistant HIV-1 to interact with the drug modified ECLs [15]. Interestingly, this study also demonstrated an increased reliance of MVC-resistant virus on His88 and His181, although not to the same degree that we saw for 17-Res and 24-Res Envs. The requirement of all the presently characterized MVC-resistant HIV-1 Envs to maintain interactions with the drug-modified ECLs, regardless of whether they were generated *in vivo* or *in vitro*[15,51], may help explain why these Envs lack appreciable cross resistance to other CCR5 antagonists or display only narrow cross resistance.

### Potential structural alterations between gp120 and the CCR5 N-terminus distinguish MVC-resistant from MVC-sensitive Envs

To identify potential structural alterations associated with MVC resistance, we constructed full-length three-dimensional (3D) homology models of gp120s of 17-Sens, 17-Res, 24-Sens and 24-Res Envs based on the crystal structure of CD4-bound YU2 gp120 [73]. This structure is particularly useful because it has an intact V3 loop and is docked to a CCR5 N-terminal region peptide (CCR5_2-17_), and thus permits the investigation of the molecular interactions between the CCR5 N-terminus and the stem of the V3 loop and the gp120 bridging sheet. However, a limitation of homology modeling of this system is that generally only a single conformation of the V3 loop and the CCR5 residues can be considered. Therefore, we have taken a conservative approach that relies on the data inherent in the available crystal (gp120) and NMR (CCR5 N-terminal region) structures used as templates to derive the homology models. With these limitations in mind, we provide below structural interpretations of the homology models in the context of our current experimental findings.

**Figure 10:**
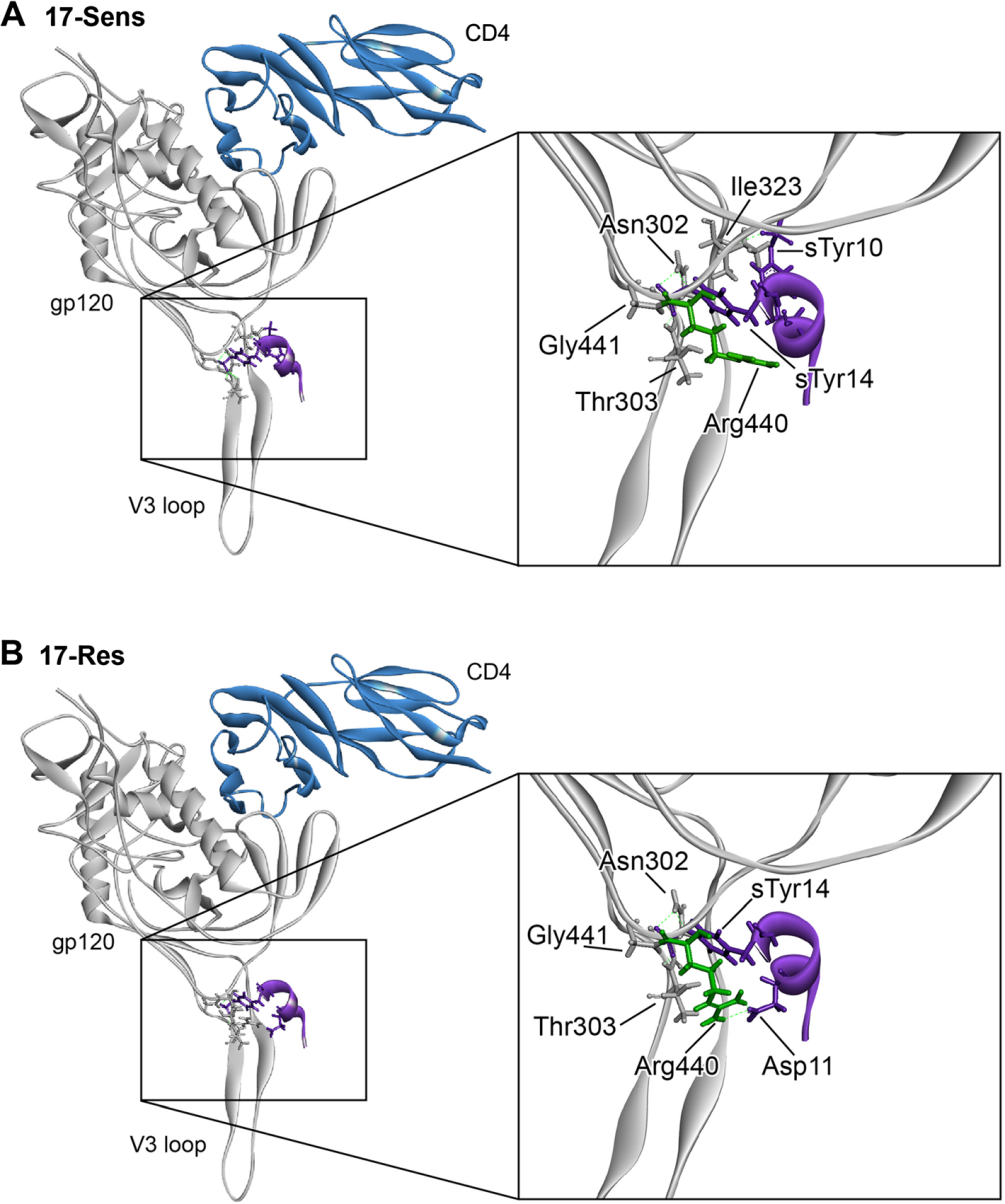
**Potential molecular interactions between gp120 of 17-Sens and 17-Res Envs and the CCR5 N-terminus.** Homology models of sensitive and resistant gp120 protein sequences were created using a crystal structure of YU2 gp120 bound to CD4 and a sulfated CCR5 N-terminus peptide mimic [73]. Models of 17-Sens (**A**) and 17-Res (**B**) are represented as grey ribbons. Amino acids involved in bonding between gp120 and the CCR5 N-terminus are shown as sticks. The Arg440 side chain is shown in green, to illustrate its potential repositioning from the bridging sheet in 17-Res Env. Purple; CCR5^2-17^ N-terminus peptide, green dashed lines; bonds.

**Figure 11:**
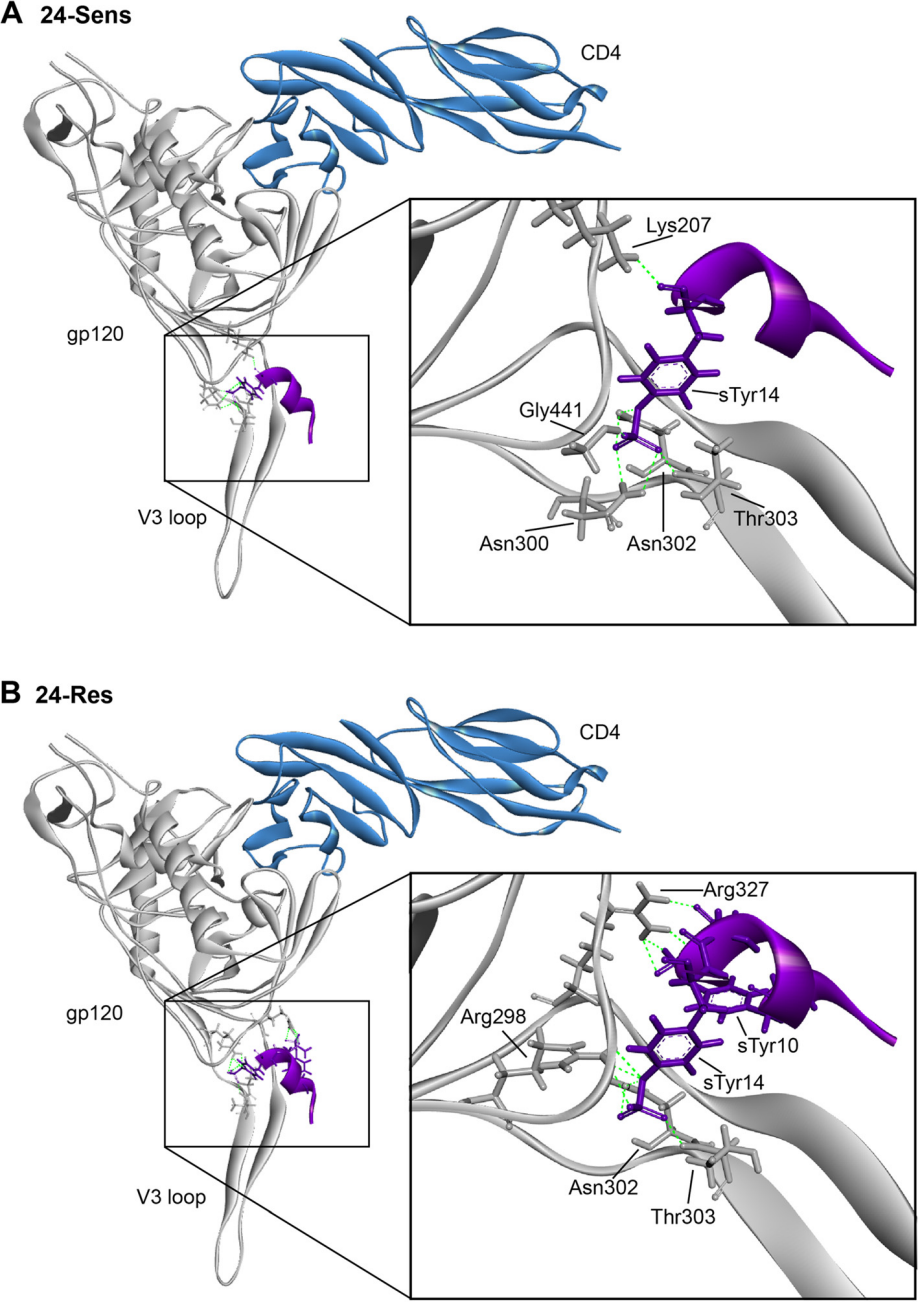
**Potential molecular interactions between gp120 of 24-Sens and 24-Res Envs and the CCR5 N-terminus.** Homology models of sensitive and resistant gp120 protein sequences were created using a crystal structure of YU2 gp120 bound to CD4 and a sulfated CCR5 N-terminus peptide mimic [73]. Models of 17-Sens (**A**) and 17-Res (**B**) are represented as grey ribbons. Amino acids involved in bonding between gp120 and the CCR5 N-terminus are shown as sticks. Purple; CCR5^2-17^ N-terminus peptide, green dashed lines; bonds.

The homology models with potential alterations in interaction networks between the CCR5 N-terminus and gp120 of MVC-sensitive and MVC-resistant Envs from subjects 17 and 24 are shown in Figures 10 and 11, respectively, and a summary of the potential bonding networks is shown in Table 2. Compared to 17-Sens gp120, the 17-Res gp120 model showed a loss of a possible hydrogen bond formed between Ile323 of gp120 and sulfated Tyr10 of CCR5, but a potential gain of two ionic (ie, electrostatic) interactions formed between Arg440 of gp120 and Asp11 of CCR5, which was associated with repositioning of Arg440 from the gp120 bridging sheet. Four hydrogen bonds between gp120 and the CCR5 N-terminus were common for both 17-Sens and 17-Res Envs, which included 2 bonds formed between Asn302 of gp120 and sulfated Tyr14 of CCR5, Thr303 of gp120 and sulfated Tyr14 of CCR5, and Gly441 of gp120 and sulfated Tyr14 of CCR5. In contrast, the 24-Res gp120 model displayed a more extensive network of potential bonds formed between gp120 and the CCR5 N-terminus compared to 24-Sens, with the only predicted interaction shared by both 24-Sens and 24-Res Envs being a hydrogen bond formed between Thr303 of gp120 and sulfated Tyr14 of CCR5. Importantly, compared to 24-Sens which possibly had one salt bridge between Lys207 of gp120 and sulfated Tyr14 of CCR5, the 24-Res model contained a more complex network of 7 electrostatic interactions between gp120 and CCR5, all formed with sulfated Tyr14 of CCR5.

**Table 2:** Potential bond networks formed by HIV-1 Envs and the CCR5 N-terminus

**Env**	**gp120 residue**	**CCR5 N-terminal peptide residue**	**Bond type**
**17-Sens**	Asn302: Hδ21	sTyr14:OH	H
	Asn302: Hδ21	sTyr14:O3	H
	Thr303:HN	sTyr14:O2	H
	Ile323:HN	sTyr10:O3	H
	Gly441:HN	sTyr14:O1	H
**17-Res**	Asn302:Hδ21	sTyr14:OH	H
	Asn302:Hδ21	sTyr14:O3	H
	Thr303:HN	sTyr14:O2	H
	Arg440:Hη12	Asp11: Oδ1	I
	Arg440:Hη22	Asp11: Oδ1	I
	Gly441:HN	sTyr14: Oδ1	H
**24-Sens**	Lys207: Hζ1	sTyr14:O	I
	Asn300:Hη21	sTyr14:O3	H
	Asn300:Hη22	sTyr14:O2	H
	Asn302:Hδ22	sTyr14:OH	H
	Asn302:Hδ22	sTyr14:O3	H
	Thr303:HN	sTyr14:O2	H
	Gly441:HN	sTyr14:O1	H
**24-Res**	Arg298:Hη21	sTyr14:OH	I
	Arg298:Hη22	sTyr14:OH	I
	Arg298:Hη22	sTyr14:O1	I
	Asn302:Hδ21	sTyr14:OH	H
	Asn302:Hδ21	sTyr14:O3	H
	Thr303:HN	sTyr14:O2	H
	Arg327:Hη11	sTyr14:O1	I
	Arg327:Hη11	sTyr14:O3	I
	Arg327:Hη12	sTyr14:O2	I
	Arg327:Hη22	sTyr14:Oδ1	I

The gp120 residues involved in interactions with the CCR5 N-terminus are numbered according to the HXB2 gp120 sequence. Bonding networks were determined as described in Methods. H, Hydrogen bond; I, Ionic hydrogen bond; Hη21, delta hydrogen atom 1; HN, backbone nitrogen; Hη11, eta hydrogen atom 1,1; Hη12, eta hydrogen atom 1,2; Hη21, eta hydrogen atom 2,1; Hη22, eta hydrogen atom 2,2; Hη1, zeta hydrogen atom 1; OH, hydroxyl; O, oxygen; O1, oxygen atom 1; O2; oxygen atom 2; O3, oxygen atom 3; Oδ1, delta oxygen atom 1.

These results suggest that the two MVC resistant Envs have structural differences involved in their altered engagement with the CCR5 N-terminus. Whilst sulfated Tyr14 seems to be a common “anchor” point for Envs from both subjects, regardless of whether they are resistant or sensitive to MVC, 24-Res Env potentially forms a more extensive network of interactions with sulfated Tyr14 compared to 24-Sens Env. 17-Res Env, on the other hand, may participate in one less interaction with sulfated Tyr14 compared to 17-Sens Env, but may have two additional electrostatic interactions with Asp11 of CCR5. These structural models help reconcile the differences in MVC resistance phenotypes and abilities to interact with MVC-bound CCR5 between 17-Res and 24-Res Envs. The more extensive bonding network formed by the “strongly” resistant 24-Res Env may result in greater affinity for the CCR5 N-terminus, and thus may underlie the highly efficient interaction with MVC-bound CCR5 that is demonstrated by the affinity profiling studies (see Figure 4). This may also explain why 24-Res Env was considerably less sensitive to inhibition by the sCCR5_1-22_ peptide than 17-Res Env (see Figure 6).

Interestingly, our molecular modeling studies suggest that none of the V3 loop changes that were shown to be the genetic determinants of MVC resistance (see Figures 1 and 3) were directly involved in the altered bonding networks formed by the resistant Envs. Instead, these amino acids may alter the conformation and integrity of the V3 loop [51], indirectly facilitating different bonding patterns between the CCR5 N-terminus and other gp120 residues. This interpretation, which is consistent with the current view of how HIV-1 can adopt altered use of CCR5 to escape CCR5 antagonists [74], provides a possible explanation as to why V3 loop mutations conferring resistance to MVC and other CCR5 antagonist are almost always strain specific.

## Conclusions

Together, the results of our study show that resistance to MVC, the only CCR5 antagonist approved for use as an antiretroviral therapy for treatment of HIV-1 infection, can develop to different extents in HIV-1 infected subjects. HIV-1 can develop comparatively “weak” or “strong” resistance *in vivo*, as a result of acquiring a relatively inefficient or efficient ability to recognize the MVC-bound conformation of CCR5, respectively, and involves strain-dependent resistance mutations that indirectly facilitate an altered interaction with the CCR5 N-terminus. Because there is no available crystal structure of gp120 bound to the CCR5 ECLs, it remains unclear as to whether the V3 loop resistance mutations are directly or indirectly involved in maintaining interactions between the MVC-resistant Envs and the drug-modified ECLs. Despite highly divergent resistance phenotypes and abilities to recognize MVC-modified CCR5, and lack of common resistance mutations, the molecular pathway to resistance by the Envs studied is remarkably similar; this pathway involves HIV-1 adopting a critical reliance on the CCR5 N-terminus, in particular on the sulfated tyrosine residues, and an altered interaction with the CCR5 ECL1 and ECL2 regions, in particular with histidine residues.

Together, our study provides new mechanistic insights into the development of resistance to MVC *in vivo*. Furthermore, the increased reliance on sulfated CCR5 N-terminus residues suggests a new avenue to block HIV-1 entry by CCR5 N-terminus sulfopeptidomimetic drugs.

## Methods

### Cells

293T cells, JC53 cells [75], NP2-CD4 and NP2-CD4/CCR5 cells [76] were maintained in Dulbecco’s Modified Eagles Medium (DMEM) supplemented with 10% (vol/vol) fetal calf serum (FCS) and 100 µg per ml of penicillin and streptomycin. CD4 selection in NP2 cells was maintained by 500 µg per ml of G418 and CCR5 expression was maintained by 1ug per ml of puromycin. 293-Affinofile cells [59] were maintained in DMEM supplemented with 10% (vol/vol) FCS, 100 µg per ml of penicillin and streptomycin, 50 µg per ml of blasticidin and 200 µg per ml of G418. Peripheral blood mononuclear cells (PBMC) were purified from the blood of healthy HIV-1 negative donors, stimulated with 5 µg of phytohemagglutinin (Sigma) per ml for 2 days and cultured in RPMI 1640 medium supplemented with 10% (vol/vol) FCS, 100 µg per ml of penicillin and streptomycin, and 20 U per ml of interleukin-2 (Roche).

### Env cloning

Infectious recombinant viruses containing the *env* gene from subjects who had failed MVC therapy with demonstrable MVC resistance and from the baseline samples of the same subjects [52,56] were used for Env subcloning. To characterise the *env* genes from these recombinant viruses in a reporter based system, the 2.1-kb Kpn1-BamH1 *env* fragment, corresponding to nucleotide positions 6348–8478 in HXB2, was amplified from full-length NL4.3 chimeric plasmids and cloned into the pSVIII-Env expression plasmid [77], as described previously [61,78]. 17-Sens, 17-Res, 24-Sens and 24-Res Envs were shown to be functional and able to support HIV-1 entry into JC53 cells when pseudotyped onto Env-deficient luciferase reporter viruses (data not shown). Envs were sequenced, and have been assigned GenBank accession numbers KC834602 to KC834605.

### Env mutagenesis

All gp120 mutants were synthesized by GenScript Pty. Ltd. (Piscataway, NJ, USA), and subcloned into the pSVIII-Env expression vector [79]. The authenticity of the gp120 mutants was verified by full-length sequencing.

### Production and titration of Env-pseudotyped luciferase reporter viruses

Env-pseudotyped luciferase reporter viruses were produced by transfection of 293T cells with plasmids pCMVΔP1ΔenvpA, pHIV-1Luc, and pSVIII-Env using lipofectamine 2000 (Invitrogen) at a ratio of 1:3:1, as described previously [61,78,80]. Supernatants were harvested 48 h later, filtered through 0.45 µM-pore size filters, and stored at −80°C. The 50% tissue culture infective doses (TCID_50_) of virus stocks were determined by titration in JC53 cells [75], as described previously [61,78].

### Synthesis of sulfated peptide mimics of the CCR5 N-terminus

The triply sulfated sCCR5_1-22_ peptide (MDYQVSSPIYDINYYTSEPSQK; sulfated at Tyr10, Tyr14 and Tyr15) was synthesized using Fmoc-strategy solid-phase synthesis on Rink amide resin using commercially available Fmoc-protected amino acids and a synthetic neopentyl-protected sulfotyrosine residue, as we and our collaborators have described previously for CCR3 [71,72]. In this synthesis, Cys20 was modified to serine to prevent the formation of inter-strand disulfide bonds. The crude sulfopeptide was cleaved from the resin using a solution of trifluoroacetic acid/triisopropylsilane/thioanisole/water (85:0.5:0.5:0.5 v/v/v/v) for 2 hours. Following removal of the acidolytic cleavage solution *in vacuo*, the crude sulfopeptide was suspended in MilliQ water (1 mg/mL) and sodium azide (50 equiv) was added in a single portion. The mixture was warmed to 70°C and stirred for 16 hours. The mixture was concentrated by lyophilization before purification by preparative reversed-phase HPLC (linear gradient of 0% 0.1 M ammonium acetate to 45% acetontrile in 0.1 M ammonium acetate over 60 min; R_*t*_ = 30 min). The sulfopeptide was lyophilized three times from MilliQ water until a constant weight was achieved. The sulfopeptide was produced as a white solid (55 mg, 7.5%, based on the original resin loading).

### HIV-1 inhibition assays

NP2-CD4/CCR5 cells (1×10^4^ in 100 µl) were seeded in flat-bottom 96-well plates 24 h prior to infection. PBMC (2×10^5^ in 100 µl) were seeded in U-bottom 96-well plates at the time of infection. The CCR5 antagonists MVC, VCV and TAK-779 were resuspended in dimethyl sulfoxide (DMSO). CCR5 N-terminus peptides were resuspended in DMSO. The anti-CCR5 antibody 2D7 was resuspended in phosphate buffered saline (PBS). Prior to infection the media was removed from cells and replaced with 100 µl of fresh media with five-fold dilutions of inhibitor (at a ×2 concentration) for 30 min at 37°C. The concentration range for MVC, VVC and TAK-779 was 5 µM to 0.064 nM; for CCR5 N-terminus peptides the concentration range was 100 µM to 1.28 nM; for 2D7 the concentration range was 25 µg/ml to 0.32 ng/ml. The concentration of DMSO (0.1%, vol/vol) or PBS (2.5%, vol/vol) was maintained in the untreated wells. NP2-CD4/CCR5 cells were infected with 200 TCID_50_ of Env-pseudotyped luciferase reporter viruses in 100 µl for 12 h at 37°C. PBMC were infected with 2000 TCID_50_ of Env-pseudotyped luciferase reporter viruses in 100 µl for 12 h at 37°C. Following this, the inoculum was removed and replaced with fresh media. The inhibitor concentrations were maintained throughout the subsequent culture period. NP2-CD4/CCR5 were incubated at 37°C for a total of 72 h, and PBMC were incubated at 37°C for a total of 96 h.

The level of HIV-1 entry was measured by luciferase activity in cell lysates (Promega) according to the manufacturers protocol. Luminescence was measured using a FLUOStar microplate reader (BMG). Background activity was assessed by mock-infected cells and was subtracted from all wells. The amount of luciferase in cells treated with an inhibitor was expressed as a percentage of that in untreated cells. The percentage of inhibition was calculated by subtracting this number from 100. The data were fitted with a nonlinear function, and alterations in inhibitor sensitivity were assessed by reductions in the MPI as described previously [34].

### 293-Affinofile assays and quantitative vector analysis

293-Affinofile cells were infected with Env-pseudotyped luciferase reporter viruses as described previously [51,59,65] (reviewed in [60]). Briefly, 48 populations of cells expressing different combinations of CD4 and CCR5 levels were generated by inducing cells with 2-fold serial dilutions of minocycline (0.156 to 5.0 ng/ml) and ponasterone A (0.0156 to 2.0 µM). CD4 and CCR5 concentrations were determined by quantitative flow cytometry (qFACS) as described previously [59,81]. The induced cell populations were then either left untreated or treated with 10 µM MVC for 30 min at 37°C, after which they were inoculated with 200 TCID_50_ of Env-pseudotyped luciferase reporter viruses and were analyzed for levels of HIV-1 entry as described above. In experiments using MVC-treated cells, the MVC concentration was maintained throughout the culture period. The relative level of virus entry achieved by each Env test was expressed as a percentage of that achieved in 293-Affinofile cells expressing the highest concentrations of CD4 and CCR5. For infections done in the presence of MVC, virus entry was expressed as a percentage of that achieved in untreated wells.

The relative dependence of Env-pseudotyped reporter viruses on CD4 and CCR5 expression levels was mathematically modeled using the VERSA computational platform (http://versa.biomath.ucla.edu), as described previously [51,59,65] (reviewed in [60]). With this model, viral infectivity is quantified using a single vector. The vector magnitude reflects the efficiency of viral entry, and the vector angle represents the relative dependence on CD4 and CCR5. In theoretical extremes, viruses that have the greatest possible sensitivity to alterations in CD4 expression but are not affected by alterations in CCR5 expression have a vector angle of 0°, and conversely, viruses that have the greatest possible sensitivity to alterations in CCR5 expression but are not affected by alterations in CD4 expression have a vector angle of 90°.

### Entry assays in cells expressing CCR5 mutants

NP2-CD4 cells transfected with WT or mutant CCR5 were infected with Env-pseudotyped luciferase reporter viruses as described previously [51,65]. Briefly, NP2-CD4 cells were transfected with plasmids expressing WT CCR5 or CCR5 with alternative mutations in the N-terminus, ECL1, ECL2 or ECL3 regions. The WT CCR5 plasmid was serially diluted (2-fold) to create populations of cells expressing a range of CCR5. These cells were used to create a standard curve of expression of which the expression of mutant CCR5 expressing cells could be matched to. Expression of CCR5 was determined by flow cytometry using the CCR5-specific antibodies 2D7 (BD Pharmingen) or CTC5 (R&D Systems). Cells were either left untreated or treated with 10 µM of MVC for 30 min at 37°C, after which they were inoculated with 200 TCID_50_ of Env-pseudotyped luciferase reporter viruses. Cells were cultured for a total of 72 h and in experiments using MVC-treated cells, the MVC concentration was maintained throughout the culture period. Cells were analyzed for levels of HIV-1 entry as described above, and the entry of Env-pseudotyped luciferase reporter viruses in cells expressing mutant CCR5 was expressed as a percentage of that achieved in cells expressing an equivalent level of WT CCR5.

### gp120 structural modeling

Three-dimensional protein structures of 17-Sens, 17-Res, 24-Sens and 24-Res gp120 sequences were prepared using the Discovery Studio suite, version 3.0 (Accelrys, San Diego, CA), as described previously [62,82]. The crystal structure of CD4-bound YU-2 gp120 containing the V3 variable loop docked with the nuclear magnetic resonance (NMR) structure of a sulfated N-terminal peptide of CCR5 (residues 2 to 15) (kindly provided by P. D. Kwong [73]) was used as a template. Harmonic restraints were applied prior to optimization using the Steepest Descent protocol, which incorporates iterative cycles of conjugate-gradient energy minimization against a probability density function that includes spatial restraints derived from the template and residue specific properties [83]. Hydrogen bonds between gp120 and the CCR5 peptide were modeled with the Monitor H-bonds protocol in Discovery Suite 3.0, using the distance criterion of 2.5 Å and selecting for intermolecular bonds only.

## Competing interests

BJ and MW are present and past employees, respectively, of Pfizer Global Research and Development. PRG and SRL are members of the ViiV Australia Scientific Advisory Board, and have received honoraria. PRG and SRL have received honoraria from ViiV for travel to conferences, and SRL has received honoraria from ViiV for speaking at and Chairing ViiV-sponsored events. The other authors declare that they have no competing interests.

## Authors’ contributions

MR, BL, PAR, RJP and PRG designed the experiments. MR, HS, RD, HZ, AE, MM, LG, BLW, JS, JF, NW and KC performed the experiments. BL, BJ, and MW supplied critical reagents and helped interpret the results. JS, PAR, SRL and MJC helped interpret the results. MR and PRG wrote the manuscript. All authors helped edit the manuscript and have read and approved the final version.
